# Food Preferences in Cats: Effect of Dietary Composition and Intrinsic Variables on Diet Selection

**DOI:** 10.3390/ani9060372

**Published:** 2019-06-19

**Authors:** Raúl A. Alegría-Morán, Sergio A. Guzmán-Pino, Juan I. Egaña, Valeria Sotomayor, Jaime Figueroa

**Affiliations:** 1 Departamento de Medicina Preventiva Animal, Facultad de Ciencias Veterinarias y Pecuarias, Universidad de Chile, Santiago 8820808, Chile; ralegria@veterinaria.uchile.cl; 2 Facultad de Ciencias Agropecuarias, Universidad Pedro de Valdivia, Santiago 7500908, Chile; 3 Departamento de Fomento de la Producción Animal, Facultad de Ciencias Veterinarias y Pecuarias, Universidad de Chile, Santa Rosa 11735, La Pintana, Santiago 8820000, Chile; sguzmanp@uchile.cl (S.A.G.-P.); jegana@uchile.cl (J.I.E.); valeriasotomayor.l@gmail.com (V.S.); 4 Departamento de Ciencias Animales, Facultad de Agronomía e Ingeniería Forestal, Pontificia Universidad Católica de Chile, Santiago 7820436, Chile

**Keywords:** cat, commercial diets, food selection, nutrients, climate season, sex

## Abstract

**Simple Summary:**

Cats tend to retain food preferences across their lifetime, but intrinsic variables could affect their diet choices so as to preserve internal homoeostasis. This work intended to study the feeding behavior of cats in relation to diet composition and some intrinsic variables (sex, age, and body weight) by analyzing data from a 10-year database of two-feeder food preference tests (2007–2017). Diet mineral components (calcium, crude fiber and ashes) affected food preferences negatively. In addition, the influence of body weight and sex manifested as lower food intake in both females and the heaviest cats (relative to bodyweight). However, only body weight affected food preferences, where the heaviest animals presented the higher preferences overall. During the cold season, animals (especially females) displayed higher food intake, whereas hot seasons increased male cat preferences for palatable diets, hence we observed an interaction between sex and climate season. In conclusion, understanding the relationship that both food composition and some intrinsic variables cats have with their diet preference could help in improving the formulation of specific pet food diets, so that these adequately satisfy the physiological and hedonic needs of domestic cats.

**Abstract:**

A ten-year database of food preference tests (*n* = 1021; period 2007−2017) was used to explore the feeding behavior of domestic cats. Principal component (PC) analysis and linear regression between food nutrients and preferences (for the most preferred diet of each test; Diet A) were performed. Intake and preference for Diet A were analyzed by intrinsic cats’ variables and climate season. The PC1 (calcium (Ca), phosphorus (P), and ash), PC2 (lipids and ether extract) and PC4 (crude fiber; CF) had borderline significance (*p* < 0.06; β = −1.42, β = −1.56, and β = 2.68, respectively). Ash and CF contents presented negative correlations with food preference (*rho* = −0.269, *p* = 0.031; *rho* = −0.338, *p* = 0.006, respectively), and Ca had borderline significance and negative correlation with food preference (*rho* = −0.241, *p* = 0.054). Body weight and sex influenced the intake of Diet A, being lower for females (β = 11.758; *p* = 0.014) and heaviest cats (β = −5.490; *p* < 0.001). However, only body weight affected food preferences, where the heaviest cats had greater preferences for Diet A. Hot season decreased food intake (β = −2,117; *p* = 0.032), mostly in females (*rho* = −3.537; *p* = 0.002). Males had greater preferences for Diet A during hot seasons (β = 10.216; *p* = 0.023) and females presented similar preferences throughout the year (*p* = 0.950). Mineral contents, body weight and sex affected food intake and preferences of cats under the influence of climate season, probably explained by adaptive changes in food detection.

## 1. Introduction

Domestic cats (*Felis silvestris catus*) display feeding behavior that stems from specific nutritional requirements [1,2,3] associated to their strict carnivore physiology, such as high levels of total crude protein linked to the presence of specific amino acids in the diet (i.e., arginine and taurine), vitamins A, D, and complex B, as well as arachidonic acid [4,5,6]. Usually, cats eat small portions throughout the day mimicking a feeding rhythm pattern that is typical of their wild cat ancestors (*Felis silvestris lybica*), who hunted small-sized prey [5,7]. Cats choose their diets based on smell, taste, temperature, and texture [6,8,9] up to the point of self-regulating consumption of certain kinds of foods to ensure an adequate intake of certain nutrients, hence balancing their diets themselves [10,11]. In this regard, cats are innately drawn to foods with a strong umami flavor, which is typically linked to a high concentration of amino acids [5].

Usually, cats show little interest in eating foods that are rich in sugar and some studies have confirmed that they do not perceive the sweet taste [5]. Hence, cats forgo sweet foods and limit their energy intake to less than 300 kJ/day, and in case of consuming high amounts of these nutrients, they typically vomit and display diarrhea afterwards [12]. Similarly, domestic cats and their ancestors had a much higher threshold (>0.05 M) than other species in regard to salt in food, which seems to fit with their nutritional requirements of sodium chloride that are covered by the high content of salt present in their regular carnivore diet [4,5,12]. Cats can react differently to new ingredients in their diet depending on their origin, experience, and nutritional status, ranging from caution and neophobia so to avoid possible intoxication or harm [13], to neophilia that results in greater intake of new and less readily available diets [14,15]. The latter situation has been observed in both wild and farm cats, helping them to broaden their food variability and meet their total nutritional requirements [14,15].

The pet industry constantly performs tests to assess food preferences in cats with the intention of understanding the variability of their choices when offered a wide range of flavors, ingredients or presentations. Furthermore, the industry is also trying to offer a range of food products catering to nutritional and hedonic requirements of cats according to intrinsic variables, such as age, breed, reproductive status, weight, or even specific health conditions [5]. However, few scientific studies have explored how intrinsic variables might alter the magnitude of preferences for different commercial cat diets [5]. Cats usually are able to control their daily calorie intake even if they are offered free access to commercial diets [16,17]. Nevertheless, obese cats cannot self-regulate their calorie intake, displaying changes in their feeding patterns that are probably due to an altered perception of macronutrients, as well as a deregulation of other physiological ingestion mechanisms that have been described for humans [6]. In the case of humans, their tolerance and preference for high-fat foods is influenced by the fact that people with a normal body mass index (BMI) have a lower threshold for detecting lipids than people whose BMI is higher [18]. When considering other variables, such as sex, researchers have found that males present lower thresholds for hunting behaviors than females, and when males are neutered, they increase their latency for attacks [19]. Meanwhile, both cats and humans experience physiological changes in regard to their feeding behavior that stem from their age, such as a decreased digestive capacity, less motor activity, and lower metabolic requirements [4,6]. Age can also affect reward areas in the brain, resulting in diet acceptance differences due to individual changes in palatability perception [20,21]. Similarly, age could also impact on olfactory and gustatory systems that trigger food preference changes and cautious feeding behavior in cats [22]. Given this background, the current study intends to explore how the preference for commercial diets could be explained by either nutritional components of the diets or by intrinsic variables of cats.

## 2. Materials and Methods 

A database covering 10 years of cat food preferences (2007−2017) was provided by the Research Centre for Pet Feeding Behaviour (FAVET–Faculty of Veterinary and Animal Sciences, University of Chile), located in the metropolitan region of Chile (Santiago city, 34°21′S, 71°18′W), and then used to analyze the effect of diet composition and intrinsic variables of cats on their food intake and preference for commercial diets. Experimental procedures were approved by the bioethical committee of “Facultad de Ciencias Veterinarias y Pecuarias”, Universidad de Chile (N°042013).

### 2.1. Animals and Housing 

A total of 1021 preference tests using 24 different mongrel domestic cats (Felis silvestris catus) (9 male and 15 female) were used in this study over the complete period (2007–2017). An average of 15 ± 2 animals was maintained per year at the Center. These animals were sourced from local shelters at an average age of 6 months and then submitted for clinical examination by veterinarians of the Clinical Sciences Department of FAVET before being registered as experimental animals. Once housed, they were trained for 3 to 6 months to ease their adaptation to the experimental procedures. During this period, they were adapted to 2-feeder preference trials with highly palatable foods in both feeders to stimulate the intake of the two options. Health of cats was assessed prior to the start of the experiments through clinical, hematologic and biochemical serum profiles, and all cats that participated in preference trials were considered healthy. The age of the cats ranged from 1 to 13 years old and females usually represented a greater percentage of the total animals. Cats were housed individually in single kennels that were split into inner and outer areas of 94 × 90 × 105 cm and 94 × 90 × 90 cm, respectively.

Cats had access to a sandbox and fresh water on the outer area of each kennel, while on the inner area animals were fed a single ration (3% of their body weight) of a commercial diet (daily at 09:30 a.m.), as well as ad libitum access to water and a comfortable bed. This ration was adjusted monthly accordingly to body weight records of each cat to ensure their body condition remained stable. Environmental conditions were partially controlled by the Centre by means of door and window management. However, average temperature varied across different seasons during the year, ranging between a maximum of 22 °C in summer and a minimum of 7.7 °C in winter [23]. Animals had access to a common playground area (300 × 375 × 480 cm) for 1 h each day, provided with environmental enrichment objects, where they could exercise. Additionally, veterinary staff from FAVET regularly checked the animals and performed clinical examinations to assess their health status. In general, cats ended their participation in experimental trials when they reached 8 years of age (with some exceptions) or when they presented diseases not compatible with the preference tests or that could alter their results.

### 2.2. Procedures to Determine Food Preferences and Database

Food trials comprised a series of two-bowl food preference tests that were performed within each kennel to assess the variability of their nutritional, physical, and/or organoleptic properties. Each trial lasted 4 days (1 test/day) in which diets were counterbalanced in position (left or right) to diminish the effect of possible side bias over preferences. Every test began on the afternoon (02:00 pm) and ended on the next morning (10:00 am). Each cat was individually tested over this 20-hour period by offering two different diets, each on one bowl of the same color, shape, and size. Rations offered were adjusted according to their body weight (3%) at all times, whether animals were participating in a trial or not. On test days, cats were fed only the experimental diets and water was freely available for them. Feeders were weighed at the beginning and the end of each test, to estimate the amount of food that the animals consumed from each diet. In order to better equate intake across animals and diminish size effects, consumption was subsequently corrected by their metabolic weight (total consumption/body weight^0.75^). As for the nutritional composition of the experimental diets, these were subjected to a proximate chemical analysis, acid hydrolysis, and a calorimetric pump (IKA^®^ calorimetric system C 2000, Wilmington, NC, USA). Diets were analyzed following standard Association of Official Analytical Chemist (AOAC) methods [24] for content of moisture (method 945.15), crude protein (CP, Kjeldahl method 945.18, N × 6.25), ether extract (EE, method 945.16), ash (method 920.153), crude fiber (CF, method 962.09), total lipids (LIP, method 922.06), calcium (Ca), phosphorus (P), dry matter (DM), nitrogen free extract (NFE) and metabolizable energy (ME). 

With the objective to homogenize and compare different studies performed across the whole period (2007–2017) without having the same standard diet across tests, the most preferred diet in each test was labeled as Diet A, whereas the less preferred one was labeled as Diet B, and the degree of preference was expressed as the relative consumption of Diet A over Diet B + Diet A allowing evaluation of the effect of variables on the selection of the most preferred diet (see Equation (1)). To this end, our data for consumption and preference of Diet A were sorted by sex, age, and body weight of each cat, as well as the current climate season at the time of performing each test.

(1) $$\frac{\mathrm{Intake}\;\mathrm{of}\;\mathrm{Diet}\;A}{\mathrm{Intake}\;\mathrm{of}\;\mathrm{Diet}\;A+\mathrm{Intake}\;\mathrm{of}\;\mathrm{Diet}\;B}\;\times 100$$

### 2.3. Statistical Analysis

#### 2.3.1. Effect of Nutrient Composition over Cats’ Food Preferences

The evaluation of how nutritional components of diets (DM, CP, CF, EE, NFE, ash, Ca, P, LIP and ME) may explain food preferences and if the components were grouped, was carried out using a principal component analysis (PCA) and ‘devtools’ and ‘ggbiplot’ packages of the statistical software R [25]. All nutritional components were expressed as the net difference between Diet A and B (only metabolic energy was expressed as the percentage difference between diets). After the PCA and multivariable linear regression was performed confronting the most important variables grouped in the PCA and cats’ preferences by using R statistic software. Spearman correlations were performed with the nutritional elements that represented the greatest variability within the significant principal component (PC) obtained from the prior analysis. 

#### 2.3.2. Effect of Intrinsic Variables Over Cats’ Food Preferences

Intrinsic variables effects on intake and preference of Diet A was examined using linear mixed models, also known as variance component models, which are characterized by the consideration of the nested structure of the data, providing a chance to decompose variance into a number of different components which can then be given a useful interpretation [26,27,28]. The intrinsic variables recorded in this study correspond to one categorical variable, sex (male or female); body weight was registered as a continuous variable. Season was also recorded, considering cold season as all the experiments performed between March 21 and September 20 (autumn and winter seasons in the southern hemisphere) and hot season as all the experiments performed between September 21 and March 20 (spring and summer seasons in the southern hemisphere); this variable was also recorded as each one of the traditional seasons (summer, autumn, winter, and spring) according to the southern hemisphere dates. Two dependent variables were analyzed (intake and food preference), leading to the generation of two models to determine the effect of the registered variables over intake and preference of Diet A in cats. Cat variable was treated as a random effect, while the rest of the variables were treated as fixed effects. The proposed model to explain intake and preference is:

(2) $$Y_{km}=\beta_{0}+\beta_{n}+b_{k}+\varepsilon_{m\left(k\right)}$$

where *Y_km_* corresponds to the intake (corrected for metabolic weight) or to Diet A preference, *β*_0_ is the intercept, *β*_n_ is the vector of fixed effects, *b*_k_ corresponds to the random effect of the kth cat and ε_m(k)_ is the error term or the elements that were not included in the analysis. This model assumes that random effects and errors are independent at the nesting level and follow an approximately normal distribution, with means equal to zero and variance equal to σ^2^. The Likelihood Ratio Test (LRT) was used to select fixed effects from the initial model (full model), removing variables whose regression coefficients were not significant (*p* > 0.05) using a stepwise backward elimination procedure [29]. An adjustment was made for potential confounding factors, being these factors retained in the final model [26]. Dummy variables were used for the case of categorical variables (e.g., sex), leaving one level as reference, and all the regression coefficients from those levels of categorical variables must be interpreted with respect to its reference level. For the estimation of possible differences between levels of a variable, an adjusted mean comparison test was performed, using the Di Rienzo, Guzmán and Casanoves test with a significance level of *p* < 0.05 [30]. A borderline significance was considered when *p*-values were between 0.1 and 0.05 [31]. All the analyses were carried out using RStudio and the statistical software R [25], plus ‘lmerTest’, ‘mgcv’, and ‘e1071’ packages.

## 3. Results

### 3.1. Effect of Nutrient Composition on Cats’ Food Preferences 

**Figure 1:**
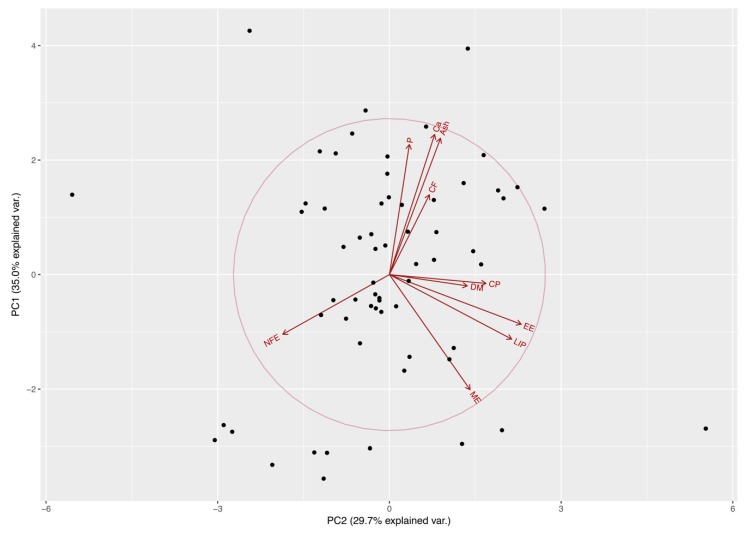
Distribution of food preference of kennel cats, on the first two principal components extracted from estimate contents of dry matter (DM), crude protein (CP), crude fiber (CF), ether extract (EE), nitrogen free extract (NFE), ash, calcium (Ca), phosphorus (P), total lipids (LIP) and metabolizable energy (ME) by proximal chemical analysis, acid hydrolysis, and calorimetric pump food decomposition. Space distribution of diet variables according to components 1 and 2, plotted as their eigenvectors.

**Figure 2:**
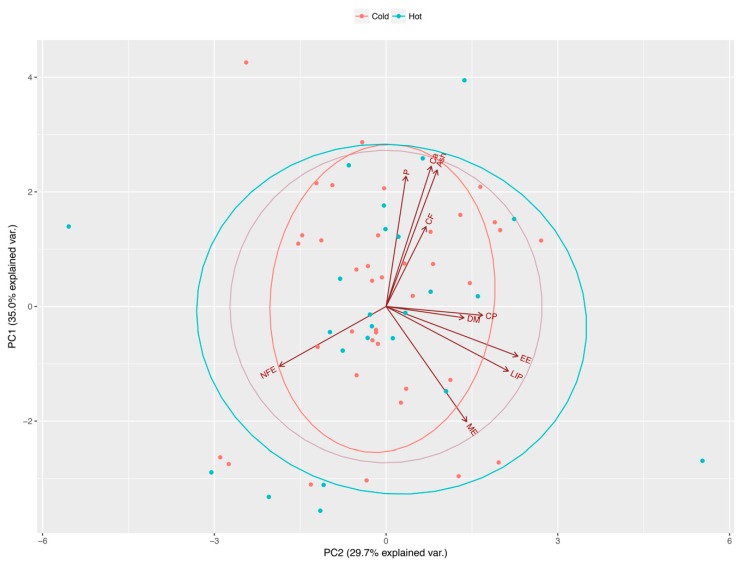
Distribution of food preference of kennel cats, on the first two principal components extracted from estimate contents of dry matter (DM), crude protein (CP), crude fiber (CF), ether extract (EE), nitrogen free extract (NFE), ash, calcium (Ca), phosphorus (P), total lipids (LIP), and metabolizable energy (ME) by proximal chemical analysis, acid hydrolysis, and calorimetric pump food decomposition. Space distribution of diet variables according to components 1 and 2, plotted as their eigenvectors and differentiated by climate season (cold and hot seasons).

Variances (i.e., eigenvalues) for the first four principal components (PC1−PC4) were greater than one (>1) and, altogether, they explained more than 85% of the variability found in the original variable (i.e., nutrients). These components allowed us to summarize our data into multivariate linear regression analyses, without losing information or minimizing such loss. In particular, the values for these components (expressed as percentages) were 34.98%, 29.70%, 13.87%, and 10.37%, respectively. Eigenvectors (Table 1) from these four components confirm that some nutrients are correlated. Four major nutrient groups were identified, specifically: a mineral component (Ca, P, and ash) in PC1, a lipid component (LIP and EE) in PC2 (see Figure 1 and Figure 2); DM and NFE in PC3; and CF in PC4. Furthermore, the linear regression analysis between principal components and the preference of Diet A by cats revealed a borderline significance for the effects of PC1 (Ca, P, and ash), PC2 (LIP and EE), and PC4 (CF) over the food preferences of cats (*p* = 0.059, *p* = 0.055 and *p* = 0.053, respectively). 

In Table 2, both positive and negative β values are observed (PC1 = −1.42, PC2 = −1.56, and PC4 = 2.68). Meanwhile, negative correlations between the contents of ash and CF with food preference appears (*rho* = −0.269, *p* = 0.031; *rho* = −0.338, *p* = 0.006, respectively). In the case of Ca, its contents in the diet had a negative correlation with food preference (*rho* = −0.241) and a borderline significance (*p* = 0.054).

### 3.2. Effect of Sex, Breed, Age, Body Weight and Season on Food Intake by Cats 

Table 3 presents the fixed effects variables that were kept in the final model for food intake and its parameter estimators. Variables that presented a significant association with cat food intake were: body weight, sex, autumn season, and the interaction term of male sex and body weight. Additionally, the interaction term of season and sex was kept in the model due to its potential role as a confounding factor.

**Figure 3:**
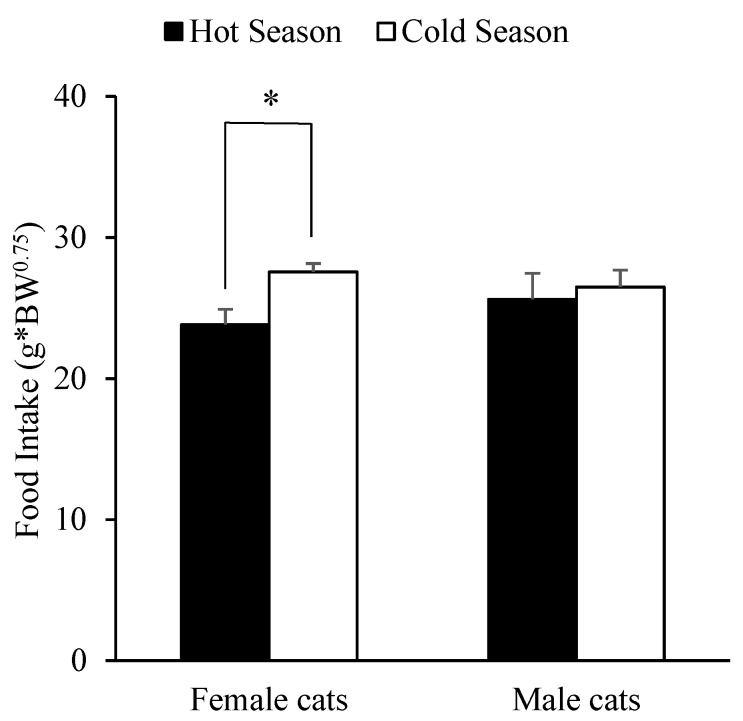
Cat’s food intake means and standard errors of most consumed diets (Diet A) after two feeder 20-h preference tests according to animal’s sex (females or males) and climate season (hot or cold). * *p* < 0.05.

In the case of body weight, there was a significant effect over food intake in cats (β = −5.490; *p* < 0.001). Specifically, we observed that food intake (corrected by metabolic weight) decreased by 5.490 units for every increase of one unit of body weight. Sex also had a significant effect as males increased their food intake by 11.758 units (β = 11.758; *p* = 0.014). As for the season, dummy variables signaled winter as the reference category, where autumn was significantly different than winter (β = 7.864; *p* = 0.022). Thus, food intake (corrected by metabolic weight) increased by 7.864 units in autumn vs. winter. Also, we observed a seasonal effect on food intake when reclassifying seasons simply as either hot (summer and spring) or cold (winter and autumn), as cats ate less from Diet A during the hot season (25.84 in hot season vs. 27.96 g in cold season, SEM 0.98 g; β = −2,117; *p* = 0.032). Meanwhile, males tended to decrease food intake as their body weight increased (β = −2.187; *p* = 0.038), whereas the interaction between sex and season did not significantly affect the intake of Diet A (*p* = 0.149). In particular, females were the only sex that significantly increased their intake under cold temperatures (*p* = 0.002 vs. *p* = 0.976 in males; see Figure 3).

### 3.3. Effect of Sex, Breed, Age, Body Weight, and Season on Food Preferences in Cats 

The mixed model analysis revealed that food preferences data was not normally distributed, hence data were transformed using the decimal logarithmic function to ensure its normality for the residual analysis. This analysis revealed that the preference for Diet A was affected by only two intrinsic variables: body weight and sex. The first one, body weight, had a complex behavior that evidenced a non-linear relation with food preference. In particular, at least two slope changes were detected, hence three relations could be observed between food preference and body weight, and clearly indicated that heaviest cats did present a greater preference for Diet A. Specifically, significant results for the first (β = −2.921 and *p* = 0.045) and third slopes (β = 1.165 and *p* = 0.001) were observed. As for the sex variable, our analysis revealed (Table 4) that males did not prefer Diet A as much as females (β = −8.419 and *p* = 0.023).

**Figure 4:**
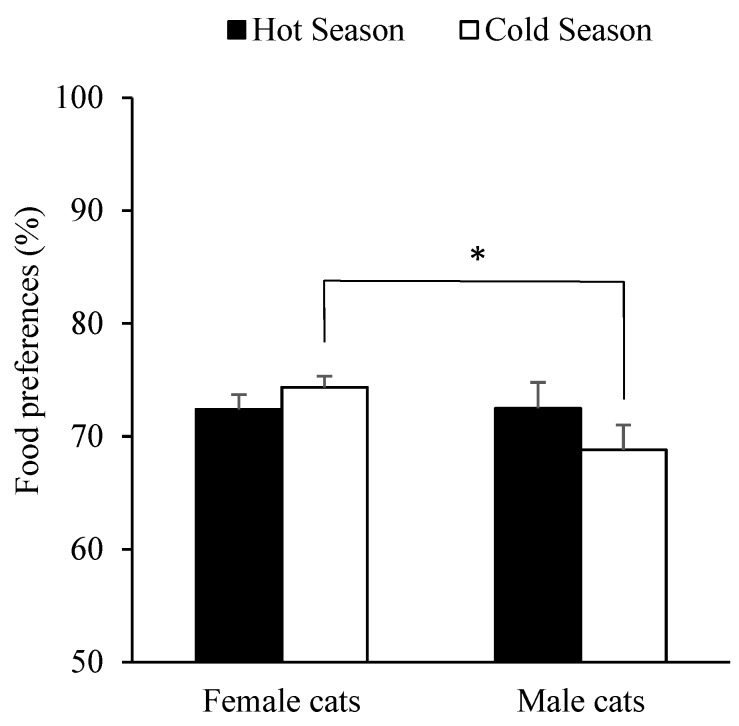
Cat’s food preferences mean and standard errors of most consumed diets (Diet A) after two feeder 20-h preference tests according to animal’s sex (females or males) and climate season (hot or cold). * *p* < 0.05.

Meanwhile, the season of the year affected food preference for Diet A, as it decreased in summer (β = −10.027 and *p* < 0.001) when it was compared against data for winter (via dummy variables). Also, the sex*season interaction did have an effect over food preference for Diet A, as males preferred Diet A more often during summer (β = 10.216 and *p* = 0.023), whereas females presented similar degree of preference for Diet A throughout the year (*p* = 0.950). The interaction between sex and season when food preferences were reclassified as either hot (summer and spring) or cold (winter and autumn) is expressed in Figure 4. 

## 4. Discussion

Cats can accept or reject some diets due to their ability of regulating their food intake according to their nutritional requirements [4,6,9,10,11]. Interestingly, the effect of intrinsic and extrinsic variables on their feeding behavior has been poorly studied [5,32], but understanding how these variables may influence food preferences in cats is highly relevant for the development of novel commercial diets that cater to specific populations and physiological conditions [32].

In this study, we assessed the effect of nutritional components on food preferences in cats and found a negative correlation with mineral components (Ca, P, and ash). In this regard, it is worth noting that the content of Ca and P in most diets used for the preference tests were higher than the percentages recommended by The Association of American Feed Control Officials [33], hence it is possible that cats had chosen diets with lower contents of these minerals because that might avoid metabolic imbalances that could adversely affect their health [1]. For instance, maintaining P and Ca within physiological levels (0.5%−0.9% and 0.6%−1.0%, respectively) in cat diets should avoid potential health problems, such as oxalate uroliths [34] or nutritional hyperparathyroidism. In addition, high calcium levels in cat diets can lead to depression, weakness, muscle spasms, anorexia, polyuria, and cardiac arrhythmias due to hypercalcemia [35]. Our results also suggest that dietary fiber decreases cats’ food preferences. Although fiber content is a necessary for a regular intestinal transit in mammals, it is negatively correlated with the nutritional content of diets intended for non-ruminants and cats may reject high inclusions on their diets. On the other hand, though an important benefit of fiber is its role in regulating body weight in animals that tend to overeat [36], diets that contain high levels of soluble fibers may cause diarrhea [37], which could also result from a poor choice of fiber source, as cats do not base their diet in the consumption of carbohydrates in large quantities [36]. 

Interestingly, the consumption of preferred diets was affected by body weight and sex in cats, but not by their age. In the case of body weight, the lower consumption of Diet A (preferred diet) that was observed in heavier animals could stem from the fact that a smaller body surface area would generate a lower specific metabolic rate (mL of O_2_ g^−1^h^−1^), reflecting a lower requirement of calories per unit of weight to maintain their daily energy requirements. Another interesting feature of the data is that heavier cats could prefer Diet A more due to some perception changes for certain macronutrients, which may arise due to failures in the physiological regulation of food intake (e.g., leptin resistance), as it has been reported for obese or overweight humans and rats [38,39], as well as due to a higher tolerance threshold for high fat diets [6,18]. 

As for the effect of sex, it manifested as a greater consumption of Diet A by male cats and it could relate to hormonal differences. In this regard, some studies have shown that androgens have anabolic effects in rats and humans, which caused greater muscle mass development in males, as well as higher metabolic rates per unit of weight than in females [40]. On the other hand, oestrogens (particularly oestradiol) are catabolic hormones that decrease the consumption of food [38,41]. Though our results did not reveal an effect of age on consumption of Diet A, it is plausible that an age-related sensory deterioration could be countered by their eating behavior patterns, which is characterised by several small rations distributed throughout the day. This would allow food to stay for a longer time within the digestive tract and then result in an increased use of nutrients [4]. A situation that was possible to observe in this work due to the decision to perform long-term trials that lasted for 20 hours.

As for the effect of seasons, it is well documented that environmental temperature affects food consumption in mammals [42,43], and the thermal comfort zone for cats ranges from 15 to 25 °C [44]. The increased consumption of Diet A during the cold season could be explained as an adjustment of caloric intake that follows the environmental temperature, thus the higher the temperature, the lower the heat loss, which decreases the consumption of the cat [45]. Results are consistent with studies performed in Europe, which demonstrate an increase in food consumption during the cold season [46]. Though a sex*season interaction over the consumption of Diet A by cats was not observed, the season did most markedly affect food consumption in females during hot months. This could relate to the seasonally polyestrous nature of cats, which cyclically alternate pro-oestrus and oestrus stages during the hot season following the increase in light hours [47]. The importance of those two stages is that they lead to increased serum oestradiol, which in turn leads to less food consumption in females [41,47]. Some studies in rats indicated that the effect of oestradiol relates to food-intake mediators by increasing the anorexic effect of cholecystokinin, serotonin and leptin, as well as reducing the orexigenic effect of ghrelin [38,48,49]. However, the fact that females ate more during the cold season might be explained by their smaller size compared to males, thus they lose heat more easily and require a higher caloric intake to compensate for it. In this regard, some studies have found in rats and humans that females increase their consumption of energetic diets during dioestrum [48]. And though the reproductive cycle of cats is different than in those species by lacking a dioestrum stage, plasma concentrations of oestradiol in queens are lower during the anoestrus stage that they undergo during the cold season—which coincides with a short photoperiod—and such decline could then explain the increase in food consumption [47,48]. 

Regarding the effect of sex*season interaction on food preferences, we observed that during the hot season only males tended to increase their preferences for Diet A. Interestingly, when Asarian and Geary [38] worked with rats, they reported that oestradiol seems to have no effect on food preferences. However, the increased food preferences for Diet A of males during the hot season might be explained because males could need more exposure to volatile cues in order to better discriminate foods via their olfactory system [13]. This is consistent with some studies in human populations that suggest lower sensitivity thresholds to olfactory and gustatory stimuli in women as the basis for their ability to more efficiently detect low amounts of nutrients or volatile cues [50,51]. Hence, it is likely that females cats are also more sensitive to volatile cues and may not need higher temperatures to discriminate better between diets that differ on their palatability. This ability to detect volatile cues in the environment helps females avoid poisoning in complex periods such as pregnancy or lactation. Moreover, it has been studied that preferences for flavors are affected by pre and postnatal learning processes in kittens and other species, indicating that female flavor identification during the perinatal period could affect the selection of a particular diet on their offspring [52].

## 5. Conclusions 

Results suggest that body weight, sex, season, and nutritional factors may affect food preferences in domestic cats. In particular, we found that excesses in the mineral component of the diets (Ca, P, ash), as well as the contents of crude fiber have a negative effect on food preferences. On the contrary, body weight decreased food consumption but increased the preference of our cats for palatable diets (Diet A). Additionally, while males usually ate more than females, sex did not affect food preferences. As for the effect of seasons, the fact that Diet A was preferred in the cold season was due to a higher consumption by females, which could relate to their reproductive cycle. Yet males tended to prefer this diet more during the hot season, probably because higher temperatures may aid them to better discriminate palatable foods by their volatile cues. Consequently, accounting for these factors when performing food preference tests—to a homogeneous feline population and under controlled environmental conditions—would help to eliminate possible biases and to reduce data variability. Such results would more reliably reveal the food preferences and feeding behavior of domestic cats when offered commercial diets. In turn, this may improve formulation and development of cat foods adjusting to fit animal’s specific nutrient requirements as well as to an adequate palatability level increasing both, cats’ health and wellbeing.

## Figures and Tables

**Table 1:** Summary table of principal component analysis (PCA), indicating the importance of each nutritional component of diets preferred by kennel cats in food preference tests, standard deviation (SD) and the percentage of explanation of variation linked to each principal component.

Nutritional Components ^1^	Principal Component Eigenvectors									
	PC1	PC2	PC3	PC4	PC5	PC6	PC7	PC8	PC9	PC10
DM	−0.04	0.29	0.66	0.24	0.32	−0.15	−0.22	0.05	−0.31	0.38
CP	−0.03	0.35	−0.38	0.51	0.49	0.12	−0.08	0.04	−0.08	−0.43
CF	0.27	0.14	0.13	−0.66	0.59	0.10	0.26	0.03	0.09	−0.14
EE	−0.17	0.49	−0.01	−0.25	−0.38	−0.24	0.25	0.11	−0.55	−0.30
NFE	−0.20	−0.39	0.52	0.04	−0.02	0.11	−0.11	0.19	0.00	−0.68
ASH	0.47	0.19	0.15	0.03	−0.15	−0.39	−0.28	−0.57	0.21	−0.31
Ca	0.48	0.16	0.06	0.16	−0.19	−0.17	0.06	0.74	0.31	−0.01
P	0.45	0.07	0.19	0.22	−0.23	0.69	0.30	−0.20	−0.22	0.01
LIP	−0.22	0.45	0.03	−0.27	−0.20	0.46	−0.57	0.08	0.29	0.00
ME	−0.39	0.30	0.25	0.19	−0.05	−0.04	0.56	−0.17	0.55	0.01
	**Principal Component Eigenvalues**									
SD	1.87	1.72	1.18	1.02	0.74	0.54	0.39	0.29	0.21	0.08
% of Variance	34.98	29.70	13.87	10.37	5.45	2.89	1.49	0.83	0.43	0.00
Cumulative %	34.98	64.68	78.55	88.92	94.37	97.25	98.74	99.57	99.99	100.0

^1^ Principal component (PC), dry matter (DM), crude protein (CP), crude fiber (CF), ether extract (EE), nitrogen free extract (NFE), ash, calcium (Ca), phosphorus (P), total lipids (LIP), metabolizable energy (ME), and standard deviation (SD).

**Table 2:** Multivariate linear regression for selected components of Principal component (PC) analysis as they relate with food preference by kennel cats.

Variable	OR ^1^	SE ^2^	*p*-Value
(Intercept)	74.3906	1.3737	<2 × 10^−16^
PC1	−1.4203	0.7403	0.0598
PC2	−1.5687	0.8034	0.0555
PC4	2.6764	1.3598	0.0537

^1^ Odds ratio or estimation of the impact of the component over food preference; ^2^ standard error.

**Table 3:** Odds ratio and associated statistic of fixed effects included in the final intake model of kennel cats.

Variable	OR ^1^	SE ^2^	*p*-Value
(Intercept)	46.90	5.21	<0.001
Weight	−5.49	0.82	<0.001
Sex			
Male	11.76	4.72	0.014
Season			
Autumn	7.86	3.43	0.022
Spring	−5.31	3.32	0.110
Summer	2.66	4.02	0.508
Weight: Sex			
Male	−2.19	1.05	0.038
Weight: Season			
Autumn	−1.68	0.99	0.089
Spring	0.52	0.86	0.548
Summer	0.50	1.11	0.652
Sex: Season			
Male: Autumn	4.20	2.52	0.096
Male: Spring	0.97	2.40	0.686
Male: Summer	4.18	2.67	0.119

^1^ Odds ratio or estimation of the impact of each variable over food intake; ^2^ standard error.

**Table 4:** Odds ratio and associated statistic of fixed effects included in the final preference model of kennel cats.

Variable	OR ^1^	SE ^2^	*p*-Value
(Intercept)	76.22	2.95	<0.001
Weight	−2.92	1.46	0.046
Weight ^2^*	−0.29	0.65	0.661
Weight ^3^*	1.16	0.37	0.002
Sex			
Male	−8.42	3.60	0.023
Season			
Autumn	−1.59	2.31	0.493
Spring	−0.76	2.25	0.736
Summer	−10.03	2.72	<0.001
Sex: Season			
Male: Autumn	7.62	4.32	0.078
Male: Spring	7.78	4.16	0.062
Male: Summer	10.22	4.65	0.028

^1^ Odds ratio or estimation of the impact of each variable over food preference; ^2^ standard error; * polynomial term of weight (square and cubic terms), indicating a change in the slope at different weights affecting food preference.

## References

[1] MacDonald M.L., Rogers Q.R., Morris J.G. (1984). Nutrition of the Domestic Cat, a Mammalian Carnivore. Annu. Rev. Nutr..

[2] Legrand-Defretin V. (1994). Differences between cats and dogs: A nutritional view. Proc. Nutr. Soc..

[3] Tobie C., Péron F., Larose C. (2015). Assessing Food Preferences in Dogs and Cats: A Review of the Current Methods. Animals.

[4] Peachey S.E., Harper E.J. (2002). Aging Does Not Influence Feeding Behavior in Cats. J. Nutr..

[5] Bradshaw J.W.S. (2006). The Evolutionary Basis for the Feeding Behavior of Domestic Dogs (Canis familiaris) and Cats (Felis catus). J. Nutr..

[6] Zoran D.L., Buffington C.A. (2011). Effects of nutrition choices and lifestyle changes on the well-being of cats, a carnivore that has moved indoors. J. Am. Vet. Med Assoc..

[7] Kane E., Rogers Q.R., Morris J.G. (1981). Feeding behavior of the cat fed laboratory and commercial diets. Nutr. Res..

[8] Bradshaw J.W.S., Thorne C.J., Thorne C.J. (1992). Feeding behaviour. The Waltham Book of Dog and Cat Behaviour.

[9] Hullar I., Fekete S., Andrasofszky E., Szocs Z., Berkenyi T. (2001). Factors influencing the food preference of cats. J. Anim. Physiol. Anim. Nutr. (Berl.).

[10] Hewson-Hughes A.K., Hewson-Hughes V.L., Colyer A., Miller A.T., Hall S.R., Raubenheimer D., Simpson S.J. (2013). Consistent proportional macronutrient intake selected by adult domestic cats (Felis catus) despite variations in macronutrient and moisture content of foods offered. J. Comp. Physiol. B.

[11] Salaun F., Blanchard G., Le Paih L., Roberti F., Niceron C. (2017). Impact of macronutrient composition and palatability in wet diets on food selection in cats. J. Anim. Physiol. Anim. Nutr..

[12] Beaver B.V., Beaver B.V. (2003). Feline Ingestive Behavior. Feline Behavior.

[13] Bradshaw J.W. (1986). Mere exposure reduces cats’ neophobia to unfamiliar food. Anim. Behav..

[14] Church S.C., Allen J.A., Bradshaw J.W.S. (1994). Anti-apostatic food selection by the domestic cat. Anim. Behav..

[15] Bradshaw J.W.S., Healey L.M., Thorne C.J., Macdonald D.W., Arden-Clark C. (2000). Differences in food preferences between individuals and populations of domestic cats Felis silvestris catus. Appl. Anim. Behav. Sci..

[16] Castonguay T.W. (1981). Dietary dilution and intake in the cat. Physiol. Behav..

[17] Kane E., Morris J.G., Rogers Q.R. (1981). Acceptability and digestibility by adult cats of diets made with various sources and levels of fat. J. Anim. Sci..

[18] Martínez-Ruiz N.R., López-Díaz J.A., Wall-Medrano A., Jiménez-Castro J.A., Angulo O. (2014). Oral fat perception is related with body mass index, preference and consumption of high-fat foods. Physiol. Behav..

[19] Inselman-Temkin B.R., Flynn J.P. (1973). Sex-dependent effects of gonadal and gonadotropic hormones on centrally-elicited attack in cats. Brain Res..

[20] Caine-Bish N.L., Scheule B. (2009). Gender Differences in Food Preferences of School-Aged Children and Adolescents. J. Sch. Health.

[21] Rolls E.T., Kellerhals M.B., Nichols T.E. (2015). Age differences in the brain mechanisms of good taste. NeuroImage.

[22] Horwitz D., Soulard Y., Junien-Castagna A., Pibot P., Biourge V., Elliott D. (2010). Comportamiento alimentario del gato. Enciclopedia de la Nutrición Clínica Felina.

[23] DMC Información Meteológica. http://www.meteochile.cl/PortalDMC-web/index.xhtml.

[24] AOAC (2005). Official Methods of Analysis.

[25] R_Core_Team (2016). R: A Language and Environment for Statistical Computing.

[26] Dohoo R., Martin W., Stryhn H. (2009). Veterinary Epidemiologic Research.

[27] Pinheiro J., Bates D. (2000). Mixed-Effects Models in S and S-Plus.

[28] Thrusfield M., Christley R. (2018). Veterinary Epidemiology.

[29] Heinze G., Wallisch C., Dunkler D. (2018). Variable selection—A review and recommendations for the practicing statistician. Biom. J..

[30] Di Rienzo J., Guzman A., Casanoves F. (2002). A multiple comparisons method based on the distribution of the root node distance of a binary tree obtained by average linkage of the matrix of Euclidean distances between treatment means. J. Agric. Biol. Environ. Stat..

[31] Dahiru T. (2008). *p*-Value, a true test of statistical significance? A cautionary note. Ann. Ib. Postgrad. Med..

[32] Aldrich G.C., Koppel K. (2015). Pet Food Palatability Evaluation: A Review of Standard Assay Techniques and Interpretation of Results with a Primary Focus on Limitations. Animals.

[33] AAFCO (2013). AAFCO Methods for Substaining Nutritional Adequacy of Dog and Cat Foods.

[34] Kerr K.R. (2013). COMPANION ANIMALS SYMPOSIUM: Dietary management of feline lower urinary tract symptoms1,2. J. Anim. Sci..

[35] BSAVA (2014). Manual de Medicina Felina.

[36] Laflammme D. (2010). Focus on Nutrition: Cats and carbohydrates: Implications for health and disease. Compendium (Yardley, PA).

[37] Fekete S.G., Hullár I., Andrásofszky E., Kelemen F. (2004). Effect of different fibre types on the digestibility of nutrients in cats. J. Anim. Physiol. Anim. Nutr..

[38] Asarian L., Geary N. (2006). Modulation of appetite by gonadal steroid hormones. Philos. Trans. R. Soc. Lond. Ser. B Biol. Sci..

[39] Park H.-J., Lee S.-E., Oh J.-H., Seo K.-W., Song K.-H. (2014). Leptin, adiponectin and serotonin levels in lean and obese dogs. BMC Vet. Res..

[40] Bell D.D., Zucker I. (1971). Sex differences in body weight and eating: Organization and activation by gonadal hormones in the rat. Physiol. Behav..

[41] Vester B.M., Belsito K.R., Swanson K.S., Keel T., Graves T.K. (2009). Impact of ovariohysterectomy and food intake on body composition, physical activity, and adipose gene expression in cats1. J. Anim. Sci..

[42] Lenis Sanin Y., Zuluaga Cabrera A.M., Tarazona Morales A.M. (2016). Adaptive Responses to Thermal Stress in Mammals. Rev. Med. Vet..

[43] Collin A., van Milgen J., Dubois S., Noblet J. (2001). Effect of high temperature on feeding behaviour and heat production in group-housed young pigs. Br. J. Nutr..

[44] Stella J.L., Croney C.C. (2016). Environmental Aspects of Domestic Cat Care and Management: Implications for Cat Welfare. Sci. World J..

[45] Shimada A. (2007). Nutrición Animal.

[46] Serisier S., Feugier A., Delmotte S., Biourge V., German A.J. (2014). Seasonal Variation in the Voluntary Food Intake of Domesticated Cats (Felis Catus). PLoS ONE.

[47] Stornelli M. (2007). Particularidades fisiológicas de la reproducción en felinos. Rev. Bras. Reprod. Anim..

[48] Heisler L.K., Kanarek R.B., Homoleski B. (1999). Reduction of Fat and Protein Intakes But Not Carbohydrate Intake Following Acute and Chronic Fluoxetine in Female Rats. Pharmacol. Biochem. Behav..

[49] Parker G.C., Bishop C., Coscina D.V. (2002). Estrous cycle and food availability affect feeding induced by amygdala 5-HT receptor blockade. Pharmacol. Biochem. Behav..

[50] Bartoshuk L.M., Duffy V.B., Miller I.J. (1994). PTC/PROP tasting: Anatomy, psychophysics, and sex effects. Physiol. Behav..

[51] Haase L., Green E., Murphy C. (2011). Males and females show differential brain activation to taste when hungry and sated in gustatory and reward areas. Appetite.

[52] Becques A., Serra J., Gouat P., Larose C. (2009). Effects of Pre- and Postnatal Olfactogustatory Experience on Early Preferences at Birth and Dietary Selection at Weaning in Kittens. Chem. Senses.

